# Peptide Receptor Radionuclide Therapy (PRRT): Innovations and Improvements

**DOI:** 10.3390/cancers15112975

**Published:** 2023-05-30

**Authors:** Elettra Merola, Chiara Maria Grana

**Affiliations:** 1 Gastroenterology Unit, G.B. Grassi Hospital (ASL Roma 3), Lido di Ostia, 00122 Rome, Italy; 2 Radiometabolic Therapy Unit, Division of Nuclear Medicine, IRCCS European Institute of Oncology, 20141 Milan, Italy; chiara.grana@ieo.it

**Keywords:** peptide receptor radionuclide therapy, neuroendocrine neoplasms, somatostatin receptor

## Abstract

**Simple Summary:**

This article discusses the use of peptide receptor radionuclide therapy (PRRT) as a key treatment method for advanced, unresectable neuroendocrine tumors. It covers the multidisciplinary theranostic approach, treatment effectiveness, patient outcomes, and toxicity of PRRT for neuroendocrine neoplasms. We will also examine important research, and explore new radiopharmaceuticals for the treatment of these patients.

**Abstract:**

Neuroendocrine neoplasms (NENs) are tumors originating from neuroendocrine cells distributed throughout the human body. With an increasing incidence over the past few decades, they represent a highly heterogeneous group of neoplasms, mostly expressing somatostatin receptors (SSTRs) on their cell surface. Peptide receptor radionuclide therapy (PRRT) has emerged as a crucial strategy for treating advanced, unresectable neuroendocrine tumors by administering radiolabeled somatostatin analogs intravenously to target SSTRs. This article will focus on the multidisciplinary theranostic approach, treatment effectiveness (such as response rates and symptom relief), patient outcomes, and toxicity profile of PRRT for NEN patients. We will review the most significant studies, such as the phase III NETTER-1 trial, and discuss promising new radiopharmaceuticals, including alpha-emitting radionuclide-labeled somatostatin analogs and SSTR antagonists.

## 1. Introduction: Neuroendocrine Neoplasms

Neuroendocrine neoplasms (NENs) represent a highly heterogeneous group of neoplasms with varying biological behavior. In fact, some cases have a very malignant behavior, whereas in other patients, disease may be stable for a long time even without any treatment. NENs are characterized by a gap between the low incidence (3–5 cases per 100,000 people annually) and the prevalence, as they are frequently slowly growing, and behave as chronic oncological diseases with a relatively long survival [1,2]. Several prognostic factors impact their survival, including the proliferative index (Ki-67), TNM stage and the World Health Organization (WHO) 2019 classification [3,4]. According to this classification, the definition of NENs includes all neoplasms with a neuroendocrine differentiation, characterized by immunolabeling for chromogranin A and synaptophysin. However, two different subgroups can be distinguished in terms of cell morphology, genetics, and prognosis: neuroendocrine tumors (NETs) and neuroendocrine carcinomas (NECs). NETs are well-differentiated neuroendocrine neoplasms, with cells presenting uniform nuclear features, “salt and pepper” chromatin, and only minimal necrosis. NETs are classified according to the proliferation index in G1 (Ki-67 index < 3%), G2 (Ki-67 index 3–20%), and G3 (Ki-67 index > 20%). Instead, NECs are high-grade, poorly differentiated neoplasms, with aggressive behavior, and presenting with abundant necrosis. They are further distinguished into small-cell NECs or large-cell NECs, based on the cell morphology. 

Somatostatin receptor (SSTR) expression characterizes approximately 90% of NENs. Among the five subtypes of SSTRs, NETs usually express SSTR2 and SSTR5, though different tumor types present considerable variability in expression [5]. SSTRs are primarily identified through functional imaging tests, which are usually performed at diagnosis both for disease staging and for choosing a therapeutic strategy. Among these techniques, which represent a standard procedure for whole-body imaging of NENs, octreotide scintigraphy with radiolabeled somatostatin analogs (SSAs) ([^111^In]In-DTPA-Octreotide) is limited by a low accuracy in detecting lesions with size < 1 cm and by a difficult semiquantitative analysis. The subsequent development of different radiolabeled DOTA-conjugated peptides (DOTA-NOC, DOTA-TOC, DOTA-TATE) for positron emission tomography/computed tomography (PET/CT) has represented a relevant innovation, progressively showing the ability to detect at least 30% more lesions than [^111^In]In-DTPA-Octreotide and conventional CT [6,7].

Despite international guidelines proposing therapeutic algorithms, NEN patients require personalized treatments based on disease characteristics [8]. Surgery with curative intent is always the option to prefer when feasible, but up to 80% of cases are metastatic at diagnosis and are not candidates for this approach. Moreover, data on adjuvant treatments are still insufficient for NENs. Thus, medical treatments represent the best approach for these patients, with somatostatin analogs (SSAs) frequently representing the first-line option when lesions express SSTRs. Other medical treatments, providing the possibility of multiple therapy lines, include chemotherapy, targeted drugs (such as everolimus and sunitinib), and peptide receptor radionuclide therapy (PRRT) [9]. This review will highlight the multidisciplinary theranostic approach, treatment effectiveness (such as response rates and symptom relief), patient outcomes, and toxicity profile of PRRT for NEN patients. We will examine the most significant studies regarding this treatment, such as the phase III NETTER-1 trial [10], and explore new radiopharmaceuticals, including alpha-emitting radionuclide-labeled SSAs and SSTR antagonists.

Genetic syndromes and the management of clinical syndrome (i.e., carcinoid syndrome) will not be discussed in the present manuscript.

## 2. Peptide Receptor Radionuclide Therapy (PRRT): Mechanisms of Action

Peptide receptor radionuclide therapy (PRRT) is a type of targeted radionuclide therapy which involves the systemic administration of therapeutic peptides labeled with radionuclides that selectively target cancer cells. Radiolabeled SSAs are the preferred choice for PRRT, as the receptor-peptide complex is internalized via endocytosis and the radionuclide is preferentially retained by the receptor-expressing tumor cells [11]. This process can lead to cell death, as the beta-particles released by lutetium-177 or yttrium-90 primarily cause DNA single-strand breaks. Furthermore, in addition to the direct effects of the radiation on treated cells, beta-particles can also impact neighboring cells by means of the cross-fire effect and bystander effect, enhancing PRRT efficacy. The former effect is attributed to the greater range of beta-particles compared to the cell diameter [12], while the latter refers to the induction of biological effects in cells near the targeted cells as if they were directly hit [13]. 

**Figure 1:**
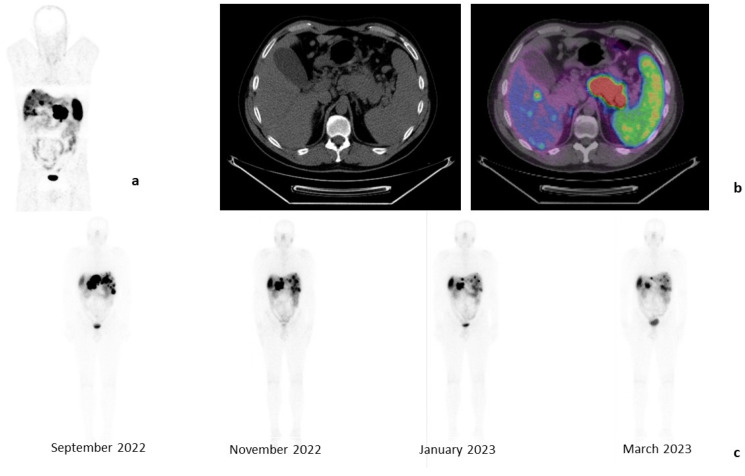
Clinical case of a 45-year-old male patient affected by a PanNET G2 (Ki-67: 6%) with liver metastases, in progression after treatment with somatostatin analogs. Baseline examination with [^68^Ga]Ga-DOTA-TOC-PET/CT (April 2022): whole-body images, anterior projection (**a**) and tomographic images of liver metastases and PanNET (**b**). Whole-body images performed 24 h after each [^177^Lu]Lu-DOTA-TATE injection, anterior projections (**c**). These images show a progressive reduction in the liver and pancreas uptake.

Additionally, patients receiving PRRT with [^177^Lu]Lu-DOTA-TATE can be easily monitored through whole-body scintigraphy performed the day after administration, using the gamma emission of lutetium (Figure 1).

## 3. PRRT in Clinical Practice

Before initiating PRRT, a baseline [^111^In]In-DTPA-Octreotide or [^68^Ga]Ga-DOTA-TOC (SomaKit TOC^®^, Advanced Accelerator Applications, Saint-Genis-Pouilly, France) is mandatory with the aim of obtaining a mapping of all SSTR-positive lesions. Candidates for PRRT should exhibit a strong SSTR expression, while diffuse hepatic and/or bone disease, as well as impaired renal function, may represent a limit to its indication. According to the ENETS Consensus Guidelines, “PRRT is a therapeutic option in progressive SSTR-positive NET with homogenous SSTR expression (all lesions are positive)” [14].

Several radiolabeled DOTA-derivatized are available. [^90^Y]Y-DOTA-TOC is currently used for locoregional treatments of liver metastases, due to its higher renal toxicity, or in some clinical trials. [^177^Lu]Lu-DOTA-TOC and [^177^Lu]Lu-DOTA-TATE are also used in PRRT, with the latter approved for gastro-entero-pancreatic (GEP-) NETs by the United States Food and Drug Administration (FDA) in 2018. The standard schedule for PRRT comprises four infusions of 7.4 GBq (200 mCi) [^177^Lu]Lu-DOTA-TATE every eight weeks, which may be extended up to 16 weeks if dose-modifying toxicity occurs [10].

Toxicity includes myelotoxicity, which can be mitigated through extracorporeal affinity adsorption treatment and is typically mild and reversible. However, up to 10% of patients may develop WHO Grade 3/4 hematotoxicity, and rarely myelodysplastic syndrome or leukemia [15,16]. Since radiopeptides accumulate in the renal interstitium, nephrotoxicity may also arise; nonetheless, it can be reduced by administering a positively charged amino acid infusion (L-lysine and L-arginine) before, during, and after the radiopharmaceutical administration, decreasing kidney radiation dose by up to 60%. This infusion may induce nausea and vomiting, and hence the concomitant administration of antiemetic drugs is recommended. Nevertheless, prior to each dose of [^177^Lu]Lu-DOTA-TATE, liver and kidney function, as well as blood-related measures, should be assessed as signs of toxicity may necessitate a longer treatment interval, reduced dosage, or even permanent cessation of the treatment [15]. 

Caution should be adopted in the case of GEP-NENs with peritoneal carcinomatosis, as this therapy has relevant limitations in controlling peritoneal disease. Furthermore, inflammation induced by PRRT may cause bowel obstruction and/or ascites in up to 22% of treated patients presenting with diffuse peritoneal disease (especially in the case of large tumor nodules) [17]. These complications may be caused by the occurrence of radiation-induced peritonitis or paralytic ileus. Similar events have been reported in the literature for other neoplasms, such as ovarian carcinomas treated with external irradiation, and may be prevented by administering a low-dose steroid starting on the day of PRRT and continuing for 2–4 weeks after therapy. The correlation between PRRT and these clinical complications, as well as the ineffective peritoneal disease control, suggest that this therapy might not be the treatment of choice in cases with diffuse carcinomatosis, and that should be reserved only for strictly selected cases with minor peritoneal involvement.

## 4. Data from the Literature

PRRT has been studied in numerous retrospective studies and single-arm clinical trials in heterogeneous patient populations that have demonstrated that radiolabeled SSAs deliver targeted radiation with a high therapeutic index to tumors that express SSTRs, thus inhibiting tumor growth in 50–70% of GEP-NETs [18]. However, the true turning point for the widespread adoption of PRRT for advanced, progressive GEP-NETs was the phase III randomized controlled trial (RCT) NETTER-1 [10]. The study demonstrated that in 229 patients affected by progressive, unresectable, midgut NETs G1–G2, the combination of [^177^Lu]Lu-DOTA-TATE and best supportive care (including Octreotide 30 mg) outperformed the monthly administration of Octreotide 60 mg alone. The progression-free survival (PFS) rates after 20 months of treatment were 65.2% and 10.8%, respectively. Following the publication of the preliminary results of NETTER-1 in the New England Journal of Medicine, the international scientific community began to recognize the potential of PRRT. Consequently, PRRT with [^177^Lu]Lu-DOTA-TATE has been approved by both the US FDA and the European Medicines Agency (EMA).

The final overall survival (OS) analysis was carried out five years after the last patient was randomized, with a median follow-up of 76 months for both groups [19]. The PRRT arm exhibited a median OS of 48.0 months, compared to 36.3 months in the control group. It is important to note that the adjusted median OS for control group patients who switched to receive PRRT was 30.9 months. 

Regarding safety, the trial demonstrated that PRRT with [^177^Lu]Lu-DOTA-TATE was well-tolerated, safe, and provided significant quality-of-life benefits compared to high-dose octreotide [20]. The concurrent administration of amino acids as renal-protective agents played a crucial role in preventing radiation damage to the kidneys. PRRT was associated with low incidences of Grade 3 or 4 hematologic toxic effects, indicating that the doses to the red marrow were not dangerously high. 

These successful results were reinforced by a meta-analysis of 22 studies investigating the efficacy of [^177^Lu]Lu-DOTA-TATE/DOTATOC in 1758 advanced/inoperable NETs [9]. The pooled disease partial response accounted for 25.0–35.0%, while the pooled disease control rate (DCR) reached 80.0%. These results proved the efficacy of PRRT as an antineoplastic therapy for GEP-NETs. 

An international consensus has then confirmed the indication for PRRT as a second-line approach for the patients with [^68^Ga]Ga-DOTA-SSA-uptake in all lesions, in NET G1–G2 at disease progression, and in selected cases of NETs G3 with all lesions being positive at [^68^Ga]Ga-DOTA-PET/CT [21].

## 5. Novel Biomarkers and Potential Role of ^18^F-FDG-PET/CT

Disease progression during PRRT has been reported in 15–30% of cases, and reliable predictive biomarkers of response to therapy are still lacking. Proposed tests include the “PRRT prediction quotient” (PPQ), which is a blood-based assay for eight genes, capable of predicting PRRT efficacy with a 97% accuracy, and the “NETest”, which boasts a 98% accuracy rate in assessing response to PRRT. Trends in NETest results correlate with PPQ predictions. However, neither test can predict toxicity [22,23]. 

The ^18^Fluorine-fluorodeoxyglucose ([^18^F]F-FDG) PET/CT may also aid in selecting patients for PRRT. As it documents the metabolic activity of tumoral lesions, and as many NENs present a low Ki-67, it has been initially reserved only for selected cases, mainly with poorly differentiated diseases. Recently, the International Consensus on the role of theranostics in NENs has expanded its application also to NECs, NETs G3, and even NETs G1–G2, with the goal of identifying the mismatched lesions ([^18^F]F-FDG-PET/CT-positive/[^68^Ga]Ga-DOTA-SSA-negative) [21]. Indeed, as up to 45% of patients referred to PRRT may exhibit heterogeneous SSTR expression, ([^18^F]F-FDG-PET/CT could differentiate GEP-NETs G1–G2 into low- and high-risk categories for poor response [24]. Chan et al. have proposed a grading system of the combined reading of SSTR-PET/CT and ([^18^F]F-FDG-PET/CT, defined as the “NETPET” score [25]. Although requiring validation in larger prospective studies, this score may represent a useful tool to apply in clinical practice for both lung and GEP-NENs [26].

Further trials aimed at assessing potential biomarkers for PRRT are currently ongoing (NCT05513469) (Table 1).

## 6. PRRT for G3 Patients

As for the application of PRRT in GEP-NENs G3, available data stem from retrospective series, suggesting a potentially active role of this therapy for highly proliferating diseases. Median PFS with PRRT reached 19 months for NETs G3 compared to 11 months for NECs with Ki-67 < 55%, and a mere 4 months for NECs with higher Ki-67 [27]. PRRT may thus be considered for GEP-NENs G3 with the following features: increased uptake on somatostatin-based imaging tests, Ki-67 < 55%, unresectable disease, reasonable performance status (Karnofski Score > 50%), and a life expectancy of at least 3–6 months [28,29,30]. At the moment, two RCTs are exploring PRRT in G2 and G3 GEP-NENs: the NETTER-2 and the COMPOSE trials, with results expected in approximately two years.

PRRT for NEC should be limited to highly selected patients, also with the support of dual tracer use involving somatostatin-based imaging tests and ([^18^F]F-FDG-PET/CT, while excluding cases with discordant ([^18^F]F-FDG-positive/SSTR-negative) lesions [29].

## 7. Future Perspectives and Ongoing Trials Regarding PRRT

Despite the significant progress with the NETTER-1 trial [10], there are still several key issues regarding PRRT that remain to be addressed. Details of ongoing trials are presented in Table 1.

### 7.1. First-Line PRRT

Although PRRT is recommended for SSTR-positive tumors after the failure of other treatments, the European Society for Medical Oncology (ESMO) guideline emphasizes considering PRRT earlier in the treatment algorithm, particularly for PanNETs [8]. The NETTER-2 study (NCT03972488) is currently assessing the efficacy of PRRT at first line adopting [^177^Lu]Lu-DOTA-TATE in combination with long-acting Octreotide in advanced GEP-NETs G2–G3 vs. high-dose (60 mg) long-acting Octreotide. This RCT includes treatment-naïve patients, patients already treated with SSAs in the absence of disease progression as well as GEP-NETs G3.

### 7.2. Neoadjuvant PRRT

Neoadjuvant PRRT for disease downstaging has been explored in a retrospective series, with the largest including 57 GEP-NETs with unresectable primary tumors, with or without liver metastases [31]. After receiving preoperative [^177^Lu]Lu-DOTA-TATE, a primary tumor was resectable in 26.3% of cases. The estimated 2-year PFS rate was 90–95%, while OS was 92.1%. Better responses were achieved in duodenal NETs, cases without regional lymph node metastases, primary tumor < 5 cm, liver lesions with size ≤ 1.5 cm and ≤3 in number, as well as an [^18^F]F-FDG uptake with a maximum standard uptake value < 5.

The NeoLuPaNET trial (NCT04385992) will assess the role of neoadjuvant PRRT in resectable Pan-NETs at a high risk of disease recurrence. The study endpoints include post-operative mortality rates and objective response rates. 

The NeoNet Trial (NCT05568017) will explore the role of neoadjuvant PRRT in unresectable or borderline resectable PanNETs G1–G2.

### 7.3. Re-Treatment with PRRT

The feasibility of re-treatment with PRRT as a salvage therapy is still under investigation. 

Severi et al. described a population of 26 progressive NETs previously treated with [^90^Y]Y-PRRT [32]. All patients had preserved kidney and hematological parameters and received 14.8–18.5 GBq of [^177^Lu]Lu-PRRT in four or five cycles. The DCR was 84.6%, the median PFS was 22 months and the toxicity was mild.

Van der Zwan et al. reported a larger experience with 181 patients affected by progressive bronchial or GEP-NETs receiving a re-(re)treatment with PRRT [33]. Median PFS was approximately 14 months, and OS was 80.8 months (95% CI 66.0–95.6). Grade III/IV bone marrow toxicity occurred in about 7% of patients after re-treatment and re-(re)treatment with PRRT, respectively.

A meta-analysis of seven studies and 414 patients affected by advanced NETs showed a median PFS of 12.52 months, and safety similar to the initial PRRT treatment [34]. These encouraging data are supported by a consensus on theragnostic in NENs, proposing PRRT rechallenge for stable disease for at least 1 year after therapy completion [21].

### 7.4. Tandem PRRT

PRRT with [^177^Lu]Lu-DOTA-TATE is expected to be less effective in large bulky lesions characterized by heterogeneous SSTR distribution, due to lower energy and a shorter particle penetration range. In contrast, yttrium-90 offers potential advantages because of its longer beta particle penetration range [35]. Based on these hypotheses, a combination of ^177^Lu-and ^90^Y-based PRRT might achieve a better response in NETs with both small and large lesions. This “tandem PRRT” approach has been investigated by several studies. Parghane et al. applied this treatment to 9 patients with tumor lesions ranging from 5.5 cm to 16 cm before PRRT, and with progressive disease after [^177^Lu]Lu-PRRT or for neoadjuvant purposes [36]. Treatment was well-tolerated, and post-PRRT [^90^Y]Y-DOTA-TATE imaging demonstrated excellent radiopharmaceutical uptake in nearly all patients. 

Seregni et al. described an objective response rate of approximately 42%, with a dramatic improvement in NET-related symptoms (i.e., pain, carcinoid syndrome) [37].

Additional data regarding 103 NET patients enrolled in a multicenter trial and treated with tandem PRRT reported a PFS of 29.9 months, with a better outcome for G1 vs. G2 cases and good tolerability [38]. 

Further data in larger, prospective populations are expected to validate these results, since unfortunately no RCTs comparing tandem PRRT vs. standard PRRT have been published so far.

### 7.5. Therapy Combination

Several studies have investigated the antiproliferative effect of therapy combinations with PRRT. The rationale for this approach is represented by the potential synergistic effect of the different treatments, but it might be counterbalanced by possible increased toxicity compared to single treatments.

In one phase II clinical trial, [^177^Lu]Lu-DOTA-TATE was combined with capecitabine and temozolomide (CAPTEM) in advanced low-grade NETs, observing a DCR of 71%, a median PFS of 31 months, while median OS was not reached [39]. Adverse events were mild to moderate, and mostly represented by nausea, thrombocytopenia and neutropenia. 

In another phase II study, [^177^Lu]Lu-DOTA-TATE was combined with metronomic capecitabine as a radiosensitizing agent in advanced, progressive [^18^F]F-FDG-positive GEP NETs with Ki-67 < 55% [40]. No renal toxicity was observed in this series, while a DCR was achieved in 85% of cases. The median PFS was 31.4 months, and the median OS was not reached. The combination with CAPTEM was also evaluated as a “sandwich” chemo-PRRT treatment [41]. The schedule was planned as follows: CAPTEM was administered within 2 weeks after PRRT, followed by a 2-week rest period. The next CAPTEM cycle was repeated similarly and followed by a one-month break, while the subsequent PRRT cycle was administered approximately 3 months later. Thus, two CAPTEM cycles were sandwiched between two PRRT cycles. With this treatment schedule, a DCR was achieved in 84% of patients, while the median PFS and OS were not reached at a median follow-up of 36 months.

As far as first-line PRRT is concerned, a series from India investigated its efficacy in combination with Capecitabine in 45 consecutive unresectable NETs, yielding promising outcomes [42]. In detail, the median PFS was 48 months, and partial response was observed in 30% of cases.

### 7.6. Therapy Sequence

Although the therapeutic landscape for NENs offers several options, determining the optimal therapy sequence aimed at achieving the best survival outcomes and tumor response remains uncertain. Numerous trials are attempting to compare different sequences in order to understand which option should be preferred based on benefits and toxicity. 

The efficacy and safety of everolimus after prior PRRT were investigated in a multicenter study that included 24 GEP-NETs [43]. Major adverse events observed with everolimus were thrombocytopenia (8.3%), fatigue (8.3%), hyperglycemia (20.8%) and elevated alanine transaminase levels (8.3%). Since median PFS was longer than observed in previous trials (13.1 months), pretreatment with PRRT might not limit the response to everolimus. 

A retrospective series of Pan-NETs pretreated with chemotherapy followed by PRRT has also been described [44], showing that previous treatment with more than one chemotherapy line was a negative prognostic factor, in contrast with survival data after resection of the primary tumor. 

The COMPETE trial is currently recruiting unresectable, progressive GEP-NETs G1–G2 to be treated with PRRT with [^177^Lu]Lu-Edotreotide vs. everolimus (NCT03049189).

### 7.7. Individualized Dosimetric Assessments

In light of the growing emphasis on personalized medicine, it is crucial to investigate the potential advantages of individualized dosimetric assessments for treatment planning and management. Garske-Roman et al. explored the impact of a dosimetry-guided treatment protocol on outcomes and side effects in 200 patients with advanced NETs [45]. Each treatment cycle consisted of 7.4 GBq of [^177^Lu]Lu-DOTA-TATE in conjunction with a mixed amino acid solution, following the standard clinical practice. Treatment cycles were repeated until the absorbed dose to the kidneys reached 23 Gy, or until other reasons necessitated stopping the therapy. In 68.5% of patients, the targeted absorbed dose of 23 Gy to the kidneys was attained after more than four cycles, resulting in significantly longer PFS and OS rates compared to patients who had to discontinue therapy before reaching 23 Gy (median PFS: 33 vs. 15 months, respectively) (median OS: 54 vs. 25 months, respectively). No major radiation-induced nephrotoxicity or bone marrow irradiation exceeding the 2 Gy threshold was observed. Given these findings and the observed intra- and interindividual variation in absorbed radiation doses for the same administered activity, we believe that the incorporation of individualized dosimetry into clinical practice may enhance the total administered activity and the number of therapy cycles, thereby significantly improving upon the standard established by the NETTER-1 trial [10]. This concept is currently being examined in several ongoing clinical trials (NCT03454763, NCT04917484).

### 7.8. Novel Radionuclides

Two additional avenues of inquiry have been proposed to enhance the effectiveness of PRRT, showing encouraging outcomes: the use of alpha-emitting radionuclides (such as ^212^Pb, ^213^Bi, and ^225^Ac) labeled with SSAs and radiolabeled SSTR-antagonists. The employment of alpha emitters is attractive due to their higher linear energy transfer, which leads to more double-strand DNA breaks, resulting in increased cytotoxicity. Moreover, the short range of soft tissue penetration (40–100 µm) of alpha emitters reduces irradiation of normal tissues, allowing PRRT to be administered as an outpatient therapy [46]. Nevertheless, it is important to consider the limited availability of alpha emitters and the scarcity of literature on the efficacy and toxicity of PRRT with alpha emitters. Currently, there is an ongoing phase I trial of [^212^Pb]Pb-DOTAM-TATE in treatment-naïve NETs (NCT03466216). Additionally, the outcomes of a first-in-human study on eight patients with progressive NETs resistant to SSAs and tandem therapy with [^90^Y]Y-/[^177^Lu]Lu-DOTA-TOC, who were treated with [^213^Bi]Bi-DOTA-TOC via either intra-arterial administration into the common hepatic artery (*n* = 7) or systemic administration (*n* = 1), can be reported. In these patients, ^213^Bi-DOTA-TOC was able to induce long-term tumor remission (overcoming resistance to beta-PRRT) while keeping nephrotoxicity and acute hematotoxicity within acceptable ranges [47].

Another promising PRRT option is represented by [^225^Ac]Ac-DOTA-TATE targeted alpha therapy, which has been investigated in a prospective series of 32 metastatic GEP-NETs who were stable or progressive on [^177^Lu]Lu-DOTA-TATE treatment. Objective response was detected in 24/32 patients, with no cases of progression or deaths in a median follow-up of 8 months, and with a reduction in circulating chromogranin [46]. Further studies are, however, needed to validate the efficacy of this therapeutic option in the case of the refractory disease to [^177^Lu]Lu-DOTA-TATE.

Despite the low internalization of antagonist-receptor complexes into tumor cells, SSTR antagonists have shown favorable pharmacokinetic characteristics compared to SSTR agonists, particularly higher tumor uptake values (due to the ability to occupy more binding sites with a lower dissociation rate), longer retention times in tumor tissue, and shorter retention times in healthy organs [48]. The most commonly used antagonist-based radiopharmaceuticals currently undergoing trials are [^111^In]In-DOTA-SST-ANT, [^177^Lu]Lu-DOTA-BASS, and **[**^177^Lu]Lu-OPS201. In a pilot study of four patients with advanced NET and chronic Grade 2 or 3 kidney disease, the latter demonstrated higher tumor uptake and longer tumoral residence time compared to **[**^177^Lu]Lu-DOTA-TATE, resulting in tumor doses up to 10 times higher [49,50]. Lastly, Baum et al. recently published the first-in-human study with the SSTR antagonist **[**^177^Lu]Lu-DOTA-LM3 in 51 patients with advanced and progressive NETs [51]. Although their study population was heterogeneous, including 69% of cases already treated with either **[**^177^Lu]Lu-DOTA-TOC or **[**^177^Lu]Lu-DOTA-TATE, a high DCR of 85% was observed, with low rates of hematological toxicity and no nephrotoxicity. Notably, baseline [^68^Ga]Ga-DOTA-TOC or DOTA-TATE PET/CT imaging showed no or low SSTR2 agonist binding in 37 of 51 patients (72.5% of patients), suggesting that agonist PRRT with **[**^177^Lu]Lu-DOTA-TOC or **[**^177^Lu]Lu-DOTA-TATE would not be feasible in these patients. Nonetheless, theranostic imaging with [^68^Ga]Ga-NODAGA-LM3 demonstrated tumor uptake greater than normal liver parenchyma in all patients. 

Lastly, numerous clinical trials have attempted to merge these two hopeful approaches by utilizing SSTR antagonists marked with alpha-emitting radioisotopes; however, the results are still in the initial stages.

It is evident that while the preliminary results of PRRT with alpha-emitting radioisotopes and/or SSTR antagonists are promising, more comprehensive studies involving larger patient cohorts and longer follow-up periods are necessary to validate these findings.

## 8. Conclusions

In summary, NENs represent a highly heterogeneous disease treated with therapeutic protocols that are not fully standardized. Based on this observation, a multidisciplinary approach is recommended for their management. Based on the available data in the literature, PRRT represents a valid and effective therapeutic option for advanced NETs, especially G1–G2 cases after SSA failure. Significant progress has been made in the past decade regarding treatments. Ongoing trials will help address the unresolved questions concerning PRRT for these patients, presenting new insights in terms of novel radiopeptides, therapy sequence and therapy combination. These findings, in conjunction with molecular profiling and the application of radiomics to understand tumor characteristics and behavior, will play a critical role in advancing precision medicine within this oncological domain.

## Figures and Tables

**Table 1:** Currently ongoing trials enrolling neuroendocrine neoplasms for PRRT treatment.

Study Name	NCT Number	Study Design	Population	Arm 1	Arm 2	Outcomes
NETTER-2	NCT03972488	Randomized, phase-III, open-label study	Unresectable GEP- NETs G2–G3, with Ki-67 10–55%, SSTR+ target lesions	[177Lu]Lu-DOTA-TATE + long-acting octreotide	High-dose long- acting octreotide	PFS; Tumor response; Duration of lesions response; Time to decline health status; Toxicity; Time to death
NeoLuPaNET	NCT04385992	Prospective, phase II, single-arm study	Resectable PanNETs, Ki-67 > 10%, size > 40 mm, SSTR+	Neoadjuvant [177Lu]Lu- DOTA-TATE → surgery		Morbidity; Mortality; Radiological response
COMPETE	NCT03049189	Randomized, phase III, open-label study	Unresectable, progressive GEP- NETs G1–G2, SSTR+	[177Lu]Lu-edotreotide	Everolimus	PFS; OS
COMPOSE	NCT04919226	Randomized, controlled, open-label, phase III study	Aggressive GEP- NETs G2–G3, SSTR+	[177Lu]Lu-edotreotide	CAPTEM, everolimus or FOLFOX	PFS; OS
P-PRRT	NCT02754297	Open-label, single-arm, phase II study	Progressive and/or symptomatic, unresectable NETs, SSTR+	[177Lu]Lu-DOTA-TATE		PFS; Tumor response; OS; Dosimetry; Safety; Quality of life
PARLuNET	NCT05053854	Open-label, single-arm, phase I study	Progressive GEP-NETs G2, SSTR+	[177Lu]Lu- DOTA-TATE + talazoparib		Toxicity; OS; Maximum tolerated dose
NeoNET	NCT05568017	Open-label, single-arm, interventional study	Unresectable or borderline resectable PanNETs G1–G2, SSTR+	Neoadjuvant [90Y]Y-DOTA-TOC (5–6 cycles, 9.25–11.1 GBq)		Operability; Circulating Biomarkers; Tumor response
	NCT03457948	Open-label, phase II, pylot study	NETs, with liver metastases, SSTR+	Pembrolizumab + (liver-directed therapy or PRRT)	Pembrolizumab	Tumor response; Toxicity; PFS
FENET-2016	NCT04790708	Open-label, single-arm study	NETs, SSTR+	PRRT (also re-treatment)		PFS; Safety; OS; Quality of life
Radio-marker	NCT05513469	Open-label, single-arm study	Advanced, midgut NETs	[177Lu]Lu-DOTA-TATE (4 cycles)		Biomarkers
	NCT05249114	Phase I study	Unresectable, progressive NETs, SSTR+	Cabozantinib 20 mg daily + [177Lu]Lu- DOTA-TATE	Cabozantinib other dosages, in 4 arms, +[177Lu]Lu-DOTATATE	Maximal tolerated dose; Tumor response
OCCLURANDOM	NCT02230176	Randomized, phase II, open-label study	Pretreated unresectable, progressive PanNETs, SSTR+	[177Lu]Lu- DOTA-TATE (4 cycles)	Sunitinib	PFS; OS; Tumor response; Quality of life
	NCT05247905	Randomized, open-label, phase II study	Unresectable PanNETs, SSTR+	[177Lu]Lu- DOTA-TATE	CAPTEM	PFS; OS; Tumor response
	NCT05475210	Open-label, phase I study	Unresectable GEP-NETs, naïve, SSTR+	[177Lu]Lu- DOTA-EB-TATE		Dose-limiting toxicity; Maximal tolerated dose; Safety; Dosimetry
	NCT03478358	Randomized, open-label, phase I study	Unresectable, progressive NETs, SSTR+	[177Lu]Lu- DOTA-EB-TATE (single dose 0.37 GBq–0.74 GBq (10–30 mCi)	[177Lu]Lu- DOTA-EB-TATE (other dosages +/− amino acids, 5 arms)	Change in [68Ga]Ga-DOTA-TATE uptake; Safety; Dosimetry
LANTana	NCT05178693	Open-label, phase I study	Progressive, metastatic NETs, Ki-67 < 55%	ASTX727 → [177Lu]Lu-DOTA-TATE		Evaluation of SSTR2 re-expression; PFS; Tolerability; Tumor response
	NCT01876771	Open-label, phase II study	Progressive NETs, SSTR+	[177Lu]Lu-DOTA-TATE	[177Lu]Lu-DOTATATE (maintenance regimen)	PFS; Tumor response; OS; Biomarkers; Quality of life; Tolerability
LUTHREE	NCT03454763	Randomized, open-label, phase II study	Progressive NENs (?), SSTR+	[177Lu]Lu-DOTA-TATE every 5 weeks for 5 cycles	[177Lu]Lu-DOTATATE every 8–10 weeks for 5 cycles	PFS; Safety; OS; Tumor response; Dosimetry
DOBATOC	NCT04917484	Randomized, open-label, phase II study	NENs, SSTR+, life expectancy > 6 mos	Dosimetry-based PRRT with [177Lu]Lu-DOTA-TOC	Standard-dose PRRT with [177Lu]Lu DOTATOC (4 cycles, 7.4 GBq)	PFS; Safety; OS; Quality of life
	NCT03466216	Open-label, single-arm, dose escalating, phase I study	Unresectable, metastatic NETs, SSTR+	[²¹²Pb]Pb-DOTAM-TATE		Dose-limiting toxicity; Maximal tolerated dose; Tumor response
ALPHAMEDIX02		Open-label, single-arm, phase II study	Progressive NETs, SSTR+, PRRT-naive	[²¹²Pb]Pb-DOTAM-TATE		PFS; response rates; time to progression; OS

GEP-NET: Gastro-entero-pancreatic neuroendocrine tumor; PFS: Progression-free survival; SSTR: Somatostatin receptor; Pan-NET: Pancreatic neuroendocrine tumor; OS: Overall survival; CAPTEM: Capecitabine and temozolomide; FOLFOX: Folinic acid, fluorouracil and oxaliplatin; PRRT: Peptide receptor radionuclide therapy; NEN: Neuroendocrine neoplasm.
